# Superficial Anaplastic Lymphoma Kinase-Rearranged Myxoid Spindle Cell Neoplasm in the Buttock: A Case Report

**DOI:** 10.3390/jpm14080858

**Published:** 2024-08-13

**Authors:** Jong-Hyup Kim, In-Chang Koh, Hoon Kim, Soo-Yeon Lim, Joon-Hyuk Choi, Kun-Young Kwon

**Affiliations:** 1 Department of Plastic and Reconstructive Surgery, Konyang University Hospital, College of Medicine, University of Konyang, Myunggok Medical Research Institute, Daejeon 35365, Republic of Korea; 2 Department of Pathology, Yeungnam University College of Medicine, Daegu 42415, Republic of Korea; 3 Department of Pathology, Konyang University Hospital, College of Medicine, University of Konyang, Myunggok Medical Research Institute, Daejeon 35365, Republic of Korea

**Keywords:** lower extremity, soft tissue neoplasm, anaplastic lymphoma kinase, tumor, CD34, S100

## Abstract

Anaplastic lymphoma kinase (ALK) is detected in both normal and oncological developmental tissues. Among ALK-related tumors, superficial ALK-rearranged myxoid spindle cell neoplasm (SAMS) is a rare, soft tissue tumor characterized by the immunophenotypical co-expression of CD34 and S100. Here, we describe a patient with this rare tumor and outline its clinical and radiological characteristics. A 28-year-old woman with diabetes, hypertension, and panic disorder presented with discomfort caused by a rubbery mass on the left buttock that had persisted for 10 years. Computed tomography revealed a multilobulated hypodense mass with small internal enhancing foci, posing challenges for the exact diagnosis of the lesion. The entire lesion was excised with clear resection margins. An 8.0 × 6.0 cm, well-circumscribed tumor with a lobular growth pattern was observed in the deep subcutaneous tissue. Light microscopy revealed epithelioid, ovoid, and spindle-shaped cells with a reticular cordlike pattern. Immunohistochemistry results were positive for S100, CD34, and vimentin. Break-apart fluorescence in situ hybridization assay results for ALK were also positive. These findings were consistent with those of SAMS. This case suggests that SAMS should be considered when identifying large nonspecific masses during clinical and imaging evaluation.

## 1. Introduction

Anaplastic lymphoma kinase (ALK), also known as the ALK tyrosine kinase receptor, has been detected in both normal and oncological tissues [1,2,3]. Among ALK-related tumors, superficial ALK-rearranged myxoid spindle cell neoplasm (SAMS) is a rare, recently reported subcutaneous tumor immunophenotypically described by the co-expression of CD34 and S100 [4]. To diagnose such a tumor, appropriate selection of molecular cytogenetic techniques, such as fluorescence in situ hybridization (FISH) testing or next-generation sequencing, is essential as conventional histological studies, which are more commonly performed, cannot provide a definitive diagnosis. The rarity of the tumor, with only a few cases reported, makes speculation further challenging and often delays the diagnostic process. Although pathological findings of SAMS have been reported, to the best of our knowledge, no reviews on its imaging characteristics, disease course, or optimal therapy are available [2]. Owing to the lack of reports on specific physical examination findings and radiological features of SAMS, presumptive diagnosis is challenging. Moreover, patients often experience heightened anxiety pre- and post-operatively. Here, we report a case of SAMS in the left buttock of a 28-year-old woman and discuss its clinical, radiological, and pathological features.

## 2. Case Description

### 2.1. Chief Complaints

A 28-year-old woman presented with discomfort caused by a rubbery mass that had developed 10 years previously on the left buttock and was incidentally discovered.

### 2.2. History of Present Illness

**Figure 1:**
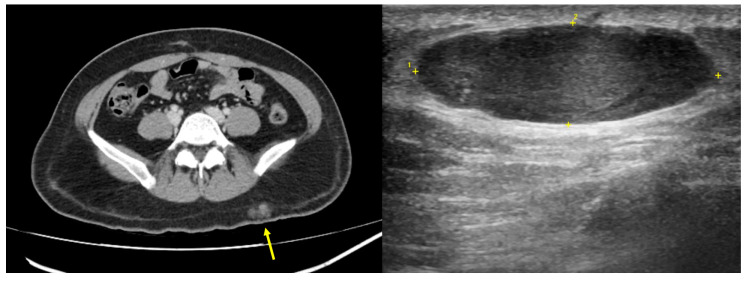
Abdominal and pelvic computed tomography (CT; (**left**)) and ultrasound (**right**) scans obtained 3 years before surgery. CT shows a well-defined, lobulated, mildly enhancing soft-tissue mass (yellow arrow) with skin thickening extending from the skin to the subcutaneous layer of the left buttock. On ultrasonography, the mass (yellow pluses indicating the margins) appears as a subcutaneous hypoechoic lesion with post-acoustic enhancement and is thus considered an epidermoid cyst.

The patient had visited the hospital 3 years earlier with the same complaint. At that time, she had undergone computed tomography (CT) and ultrasonography. CT revealed a well-defined, lobulated, mildly enhanced soft tissue mass. Ultrasonography showed a subcutaneous hypoechoic mass with a post-acoustic enhancement measuring 4.2 × 3.2 × 1.4 cm, which was most likely an epidermoid cyst (Figure 1). However, the patient refused surgical excision because of the fear of surgery. Over the past 3 years, the mass had steadily increased in size without the development of additional symptoms, prompting the patient to revisit the hospital.

### 2.3. History of Past Illness

The patient had a history of diabetes, hypertension, and panic disorder. The diabetes and hypertension were well controlled with medications such as dipeptidyl peptidase-4 inhibitors and sodium-glucose transport protein 2 inhibitors for diabetes and angiotensin II receptor and calcium channel blockers for hypertension. Moreover, the panic disorder was well managed with medications, such as serotonin–noradrenaline reuptake inhibitors, benzodiazepines, and buspirone.

### 2.4. Personal and Family History

No family history of diabetes or hypertension was reported, and the mechanism underlying panic disorder onset in the patient at age 20 was unclear. The patient did not consume alcoholic beverages or smoke. When the patient decided to receive treatment, the panic disorder was well managed, and concerns regarding surgery were resolved.

### 2.5. Physical Examination

On physical examination, the tumor was approximately 7.0 × 7.0 cm, rubbery, relatively fixed to the skin, and bluish.

### 2.6. Imaging Examination

**Figure 2:**
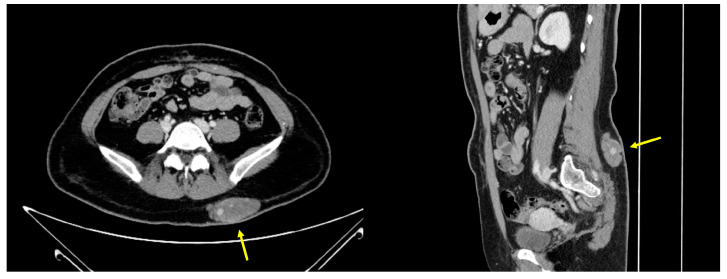
Axial (**left**) and sagittal (**right**) abdominal and pelvic CT scans obtained immediately before surgery. Both scans show a large, multilobulated, hypodense mass (yellow arrows) with internal enhancing foci in the left buttock.

The patient underwent repeat CT, which revealed a multilobulated hypodense mass including small internal enhancing foci (Figure 2).

## 3. Diagnostic Assessment

### 3.1. Macroscopy

**Figure 3:**
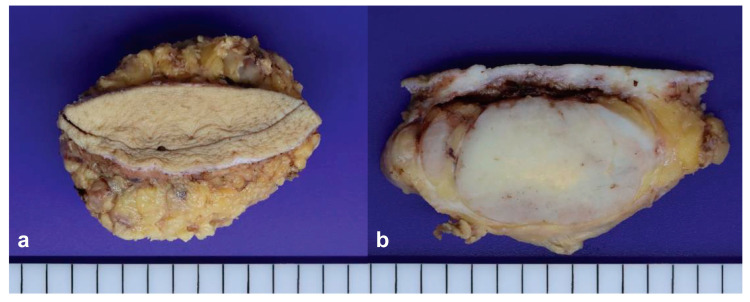
Gross findings. A well-circumscribed, yellowish-white solid tumor with a lobular growth pattern in the subcutaneous area. (**a**) Surgically excised tumor; (**b**) Cut section of the tumor.

The tumor was completely resected through a fusiform incision. A well-circumscribed yellowish-white solid tumor with a lobular growth pattern measuring approximately 8.0 × 6.0 cm was observed in the deep subcutaneous tissue adjacent to the fascia (Figure 3).

### 3.2. Histopathological Results

**Figure 4:**
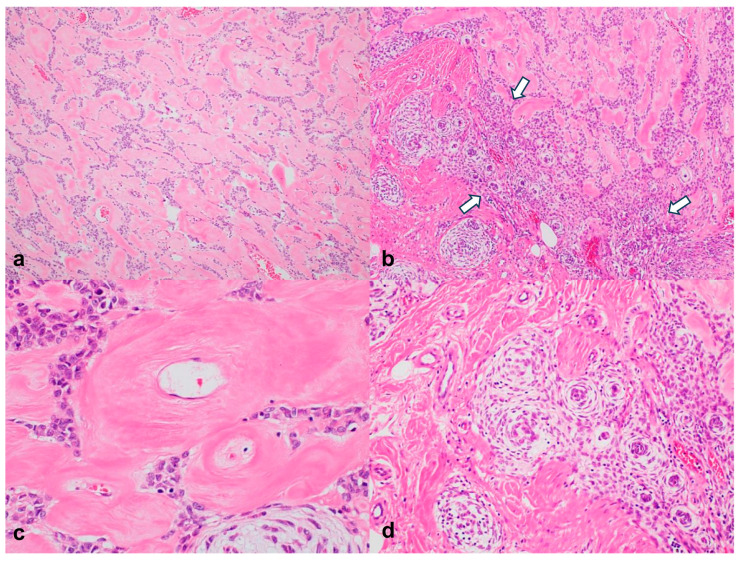
Histopathological findings. (**a**–**c**) Tumor cells showing reticular, cord (**a**), and focally hypercellular ((**b**), arrows) patterns in the prominent hyalinized stroma, as well as perivascular hyalinization (**c**). (**d**) Tumor cells arranged in striking whorls within the myxoid matrix. Hematoxylin and eosin staining; original magnification: (**a**,**b**), 100×; (**c**), 400×; (**d**), 200×.

Light microscopy revealed that the tumor comprised variable epithelioid, ovoid, and spindle-shaped cells with vesicular nuclei and marginal amphophilic cytoplasms. The tumor cells varied in structure, with reticular, cordlike, and focal hypercellular patterns. The tumor had prominent stromal and perivascular hyalinization, and the tumor cells were arranged in striking whorls within the myxoid matrix (Figure 4). No substantial pleomorphisms were observed and mitoses were rare.

### 3.3. Immunohistochemistry

**Figure 5:**
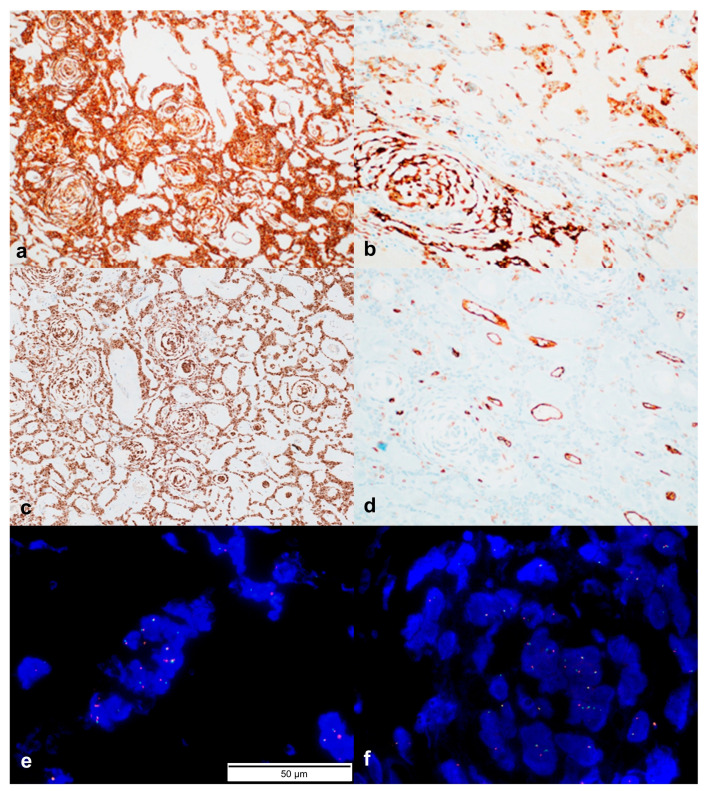
Immunohistochemical analysis of tumor cells. (**a**–**d**) Diffuse positivity for CD34 (**a**), S100 (**b**), and anaplastic lymphoma kinase (ALK; (**c**)), and negativity for CD31 (**d**). Original magnification (**a**–**d**): 100×. (**e**,**f**) Break-apart fluorescence in situ hybridization reveals ALK rearrangements.

Immunohistochemically, the tumor cells showed diffuse positivity for S100, CD34, and vimentin; focal positivity for TFE-3; and negative expression of CD31, desmin, EMA, myogenin, cytokeratin (AE1/AE3), SMA, p63, SOX10, HMB45, pan-TRK, and cytokeratin 5/6. The Ki-67-labeling index was approximately 5%. A break-apart FISH assay for ALK was performed, and ALK gene rearrangements were identified (Figure 5).

## 4. Final Diagnosis

Upon initial pathological examination of the tumor, accurate classification was challenging due to the limited histopathological and immunohistochemical staining results. Therefore, a preliminary descriptive diagnosis of “atypical round and spindle cell neoplasm with uncertain malignant potential” was made. The case was then referred to another hospital for further evaluation and diagnosis, where additional immunohistochemical staining and a FISH test were performed, revealing the presence of ALK gene rearrangement. The comprehensive results, including the histological findings, immunohistochemical co-expression of S100 and CD34, and FISH findings were consistent with those of SAMS.

## 5. Treatment

Considering the anticipated difficulty in managing the wound due to its site, the patient was hospitalized for approximately 1 week postoperatively to receive wound dressing every alternate day. A second-generation cephalosporin antibiotic was administered orally for 5 days. Considering the patient’s history of diabetes and hypertension, management included maintaining 2 h postprandial blood sugar levels below 180 mg/dL and blood pressure below 130/80 mmHg to promote rapid wound healing and reduce the possibility of hematoma formation. No additional treatment beyond wound care was required after the final diagnosis.

## 6. Outcome and Follow-Up

The patient visited the outpatient clinic periodically five times, and the wound healed completely without any complications such as wound dehiscence or infection. The patient showed stable progress without signs of recurrence for up to 9 months after surgery.

## 7. Discussion

This report presents a rare case of superficial ALK-rearranged myxoid SAMS in the left buttock, highlighting its clinical, radiological, and pathological features. The findings include distinctive imaging characteristics and histological patterns that differentiate SAMS from other subcutaneous tumors.

ALK genes have been investigated in a multifarious oncological spectrum, including ALK-positive anaplastic large cell lymphomas [5], non-small cell lung cancer [2,6,7], spitzoid neoplasms [8,9], inflammatory myofibroblastic tumors [10,11], and epithelioid fibrous histiocytomas [12]. A recent case report described scalp skin tumors resembling an immunophenotype involving bundles of spindle cells and stromal hyalinization with an associated EML4–ALK fusion [13]. A spindle cell neoplasm emerging from a deeper anatomical area, such as an intraosseous vertebra, with EML4–ALK23 fusion and immunoreactivity for CD34 and S100 has also been reported [14]. Among ALK gene fusion-related neoplasms, SAMS is a recently reported tumor distinguished by the existence of myxoid spindle cell whorls and cords with co-expression of ALK, CD34, and S100 proteins [2]. Other CD34+/S100+ subcutaneous tumors with repetitive tyrosine kinase fusions involving BRAF, RAF, and NTRK1 have also been described. In the present case, confirmation of ALK rearrangements through histopathological features provided a reasonable basis for classifying our patient’s lesion as SAMS [15].

To the best of our knowledge, no report has simultaneously described the pathological aspects, clinical features, and radiological changes associated with SAMS. When our patient first presented to the hospital 3 years previously, CT revealed a lobulated soft tissue tumor with skin thickening extending from the skin to the subcutaneous layer of the left buttock. Therefore, we performed ultrasonography, which revealed a hypoechoic mass with posterior acoustic enhancement, strongly suggestive of epidermoid cyst. However, CT performed immediately before surgery revealed that the mass had enlarged during those 3 years, with the presence of small inner-enhancing foci, complicating the accurate diagnosis of the lesion. Intraoperatively, the mass appeared well circumscribed with lobular growth, leading us to consider it a nonspecific, benign mesenchymal neoplasm. No apparent feeding vessels were indicative of hemangioma. In addition, the differential histological diagnoses included an ossifying fibromyxoid tumor, myoepithelioma, and tenosynovial giant cell tumor. Ossifying fibromyxoid tumors frequently exhibit capsular ossification and are negative for CD34 [16]. Myoepitheliomas show either nested, reticular, or solid growth, or the three variably spindled or epithelioid cells are negative for CD34 [17]. Tenosynovial giant cell tumors have small histiocytic, large amphophilic, multinucleated giant and foam cells and are negative for S100 and CD34 [18].

The clinical and radiological classification of large nonspecific masses is essential, as malignancy cannot be ruled out. According to a previous study, four of six patients did not experience recurrence, and two were lost to follow up [2], suggesting that SAMS is benign and local recurrence is infrequent.

A limitation of this study was the short-term postoperative follow up. Further studies with long-term follow up are warranted to better understand SAMS. High-quality patient education and counseling are required to mitigate the anxiety stemming from the rarity and diagnostic challenges of SAMS. These can be achieved by sharing comprehensive information from long-term follow-up studies and ensuring that patients are thoroughly informed about their condition. Such efforts will help patients manage their expectations and anxiety better, ultimately leading to improved overall outcomes.

## 8. Conclusions

Here, we present the histopathological characteristics of SAMS in the buttock, a rare tumor, along with clinical and radiological findings. As there was no evidence of malignancy on histological examination in our patient, no additional treatment was administered. However, the clinical characteristics of our patient were observed over a short follow-up period, warranting further long-term clinical observations.

## Data Availability

The original contributions of this study are included in the article. Further inquiries can be directed to the corresponding authors.
